# Organic Electrochemical Transistors (OECTs) Toward Flexible and Wearable Bioelectronics

**DOI:** 10.3390/molecules25225288

**Published:** 2020-11-13

**Authors:** Ariana Villarroel Marquez, Niall McEvoy, Amir Pakdel

**Affiliations:** 1 ISM, CNRS UMR 5255, University Bordeaux, Bordeaux INP, F-33607 Pessac, France; Ariana.Villarroelmarquez@enscbp.fr; 2 AMBER & School of Chemistry, Trinity College Dublin, D02PN40 Dublin, Ireland; nmcevoy@tcd.ie; 3 Department of Mechanical, Manufacturing & Biomedical Engineering, School of Engineering, Trinity College Dublin, D02PN40 Dublin, Ireland

**Keywords:** organic electrochemical transistors, OECTs, organic semiconductors, conductive polymers, flexible bioelectronics, wearable biosensors

## Abstract

Organic electronics have emerged as a fascinating area of research and technology in the past two decades and are anticipated to replace classic inorganic semiconductors in many applications. Research on organic light-emitting diodes, organic photovoltaics, and organic thin-film transistors is already in an advanced stage, and the derived devices are commercially available. A more recent case is the organic electrochemical transistors (OECTs), whose core component is a conductive polymer in contact with ions and solvent molecules of an electrolyte, thus allowing it to simultaneously regulate electron and ion transport. OECTs are very effective in ion-to-electron transduction and sensor signal amplification. The use of synthetically tunable, biocompatible, and depositable organic materials in OECTs makes them specially interesting for biological applications and printable devices. In this review, we provide an overview of the history of OECTs, their physical characterization, and their operation mechanism. We analyze OECT performance improvements obtained by geometry design and active material selection (i.e., conductive polymers and small molecules) and conclude with their broad range of applications from biological sensors to wearable devices.

## 1. Introduction

Emerging flexible technologies, from electrochromic devices to energy storage and bioelectronics, share as a cornerstone a combination of electron and ion transport [1,2,3,4]. Thus, “soft” organic semiconductor materials, with intrinsic flexibility and mixed conduction properties (ionic and electronic conduction), have quickly advanced to the forefront of organic electronic fields due to their broad potential applications. Among these applications are organic electrochemical transistors (OECTs) that have shown great promise as ion-to-electron transducers and amplifiers for small ion-concentration changes [5,6].

**Figure 1:**
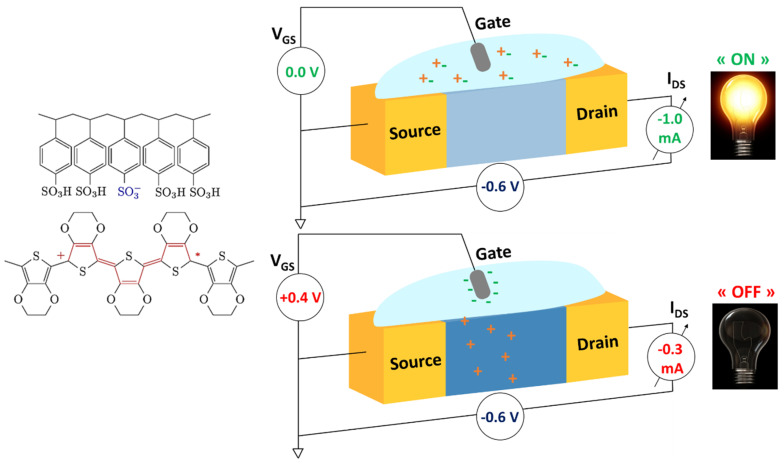
Schematic of an organic electrochemical transistor (OECT). A bias voltage (*V_DS_*) is applied between two electrodes, source and drain, which are connected by a channel made of an organic semiconductor, e.g., p-type poly(3,4-ethylenedioxythiophene) doped with polystyrene sulfonate (PEDOT:PSS). This channel is in contact with an electrolyte where a third electrode is immersed, the gate electrode, *V_G_*, (i.e., Ag/AgCl electrode). (**Top**): In the absence of a gate voltage, the channel is in its conductive (oxidized) state; this is the “on” state of the device. (**Bottom**): Application of a positive voltage results in reduction of the channel material (neutral state), switching the device to the “off” state.

The first OECT described by White and coworkers [7] was a device composed of two gold electrodes and a polypyrrole (PPy) channel. This benefited from the ambivalent character of organic semiconductor materials by conducting electrons, as well as ions. A schematic representation of an OECT is shown in Figure 1. This transistor involves a channel made of an organic semiconductor material which is placed between two electrodes, called the source and the drain. The flow of charge carriers between the source and the drain provides an electrical response, called the drain current (*I_D_*). This can be tuned by the presence of ions in the electrolyte solution, which surrounds and is in direct contact with the semiconductor material, as well as by applying a voltage (*V_G_*) at the gate electrode, which is immersed in the electrolyte solution.

The working principle of an OECT is based on doping-state changes in the semiconductor channel material due to electrolyte-ion injections which modify the electrical conductivity [8]. As a graphic example, we use an omnipresent material in organic electronics, p-type poly(3,4-ethylenedioxythiophene) doped with polystyrene sulfonate (PEDOT:PSS), to clarify this mechanism.

By the application of a positive gate voltage (Figure 1, bottom), cations (M^+^) from the electrolyte diffuse through the electrolyte due to electrostatic repulsion and penetrate into the polymer matrix. A balancing of charges with the polyelectrolyte PSS takes place, resulting in the reduction of PEDOT as shown in Equation (1).

(1) $$PEDOT^{+\;-}PSS+\;M^{+}+\;e^{-}⇌PEDOT^{0}+PSS^{-\;+}M.$$

Consequently, the drain current decreases due to the nonconductive character of this reduced state (*PEDOT*^0^). This working mode is known as “depletion mode” where the transistor is switch off by the extraction of holes from the channel [8]. Conversely, devices can also operate in “accumulation or enhancement mode” by using a p-type material which is undoped in its initial state; in this case, the OECT is in the “off” state when no gate voltage is applied due to the low availability of holes in the channel. By applying a negative gate voltage, anions penetrate into the matrix of the semiconductor material, resulting in the accumulation of holes and switching the device to its “on” state. For this reason, high stability in an oxidized and neutral form in aqueous media and high charge-carrier mobility are essential requirements for p-type channel materials in OECT devices.

For n-type materials, electrons are the main charge carriers, and they can be either extracted or injected to operate transistors in depletion or accumulation mode, respectively. When using n-type materials, the two operation modes can be achieved by applying a gate voltage with the opposite sign with respect to the p-type materials, i.e., negative gate voltage for depletion mode and positive for accumulation. Stability of the reduced and neutral forms of n-type materials is required for their use in OECT devices.

## 2. Physical Characterization: Figure of Merit (FoM), Modeling, and Performance Design

From the working mechanism (Figure 1), key aspects that govern the choice of semiconductor channel materials for OECTs can be deduced. These properties have been recognized as (1) electron mobility, responsible for the electrical conduction in the channel, (2) volumetric capacitance, which guarantees the swelling ability of the material and penetration of the ions from the electrolyte into the channel layer, and (3) ion mobility along the channel material [8,9].

**Figure 2:**
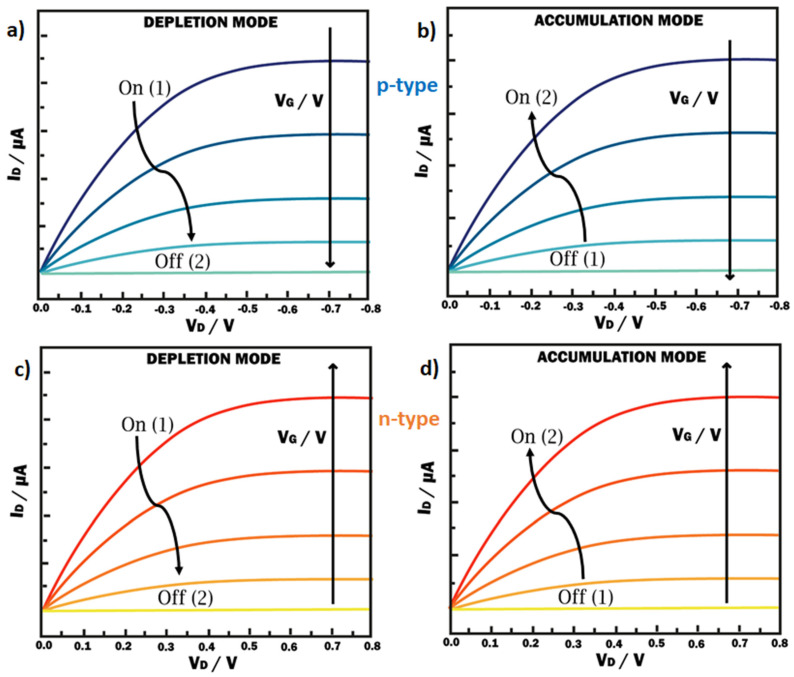
Representative output characteristics of OECT devices. For *p-type materials* (Blue, Top): (**a**) In depletion mode, under the application of a positive gate voltage (*V_G_*) cations penetrate into the polymer, dedoping the channel and switching the transistor to its “off” state; (**b**) in accumulation mode, Applying a negative gate voltage (*V_G_*) results in anions entering the channel material, switching the device to the “on” state. For *n-type materials* (Red, bottom), both modes of operation are obtained applying opposite sign of gate voltage (*V_G_*): (**c**) negative for depletion and (**d**) positive for accumulation.

The infiltration of ions into the channel and, consequently, the material’s doping extent/degree (redox state) are controlled by the applied gate voltage (*V_G_*). The drain–source voltage (*V_DS_*), i.e., the difference in potential between source and drain, determines the intensity or magnitude of the channel current (*I_D_*) observed in the drain (Figure 2). Thus, I_D_ is proportional to *V_DS_* until the achievement of a saturation state. Consequently, the output characteristics would show a decrease of the drain current when *V_G_* is increased in the case of “depletion mode” operation (Figure 2a). On the contrary, an increase of drain current would be observed in “accumulation mode” when anions are injected into the channel (Figure 2b). Applying a negative gate voltage will induce a higher hole mobility in the semiconductor channel.

**Figure 3:**
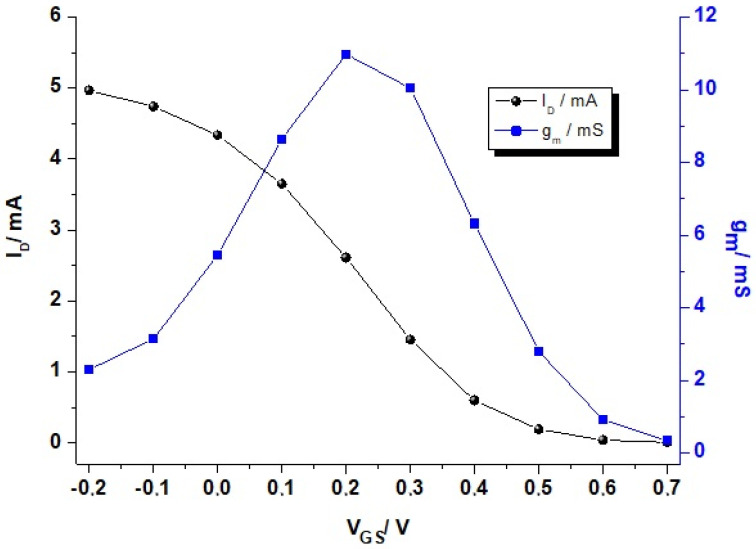
Representative transfer curve and transconductance (*g_m_*) of an OECT in depletion-mode operation. *Black curve*: Transfer curve (spheres dots) with an “on” state at *V_G_* = −0.2–0 V and “off” state at *V_G_* = +0.5–0.6 V. *Blue curve*: Transconductance (square dots) of the transistor with maximum *g_m_* at +0.2 V. Conditions: *V_DS_* = −0.4 V, material: PEDOT:PSS, dimensions: L = 1 µm, t = 100 nm.

OECTs can also be used as an amplifier; they can convert low signals in gate voltage into larger changes in drain current. The transfer curve (*I_D_* vs. *V_G_*, for a constant *V_DS_*) reflects the relationship between the drain current and the gate voltage applied. This can be used as a simple means of visualizing a transistor switching from its “on state” (high drain current) to the “off state” (very low current) in depletion mode (black curve, Figure 3). The efficiency of this amplification, i.e., ion-to-electron transduction, introduces one figure of merit (FoM) for OECTs, the transconductance (*g_m_*, blue curve, Figure 3).

The transconductance is the modulation of the current in the semiconductor material (channel) in relation to the variation of the gate potential drain (Figure 3). As expressed in Equation (2), this relates the change in current (∂*I_D_*) to the change in gate voltage (∂*V_G_*); thus, mathematically, it reflects how steep the slope of the transfer curve is and, with this, the amplification power of the OECT.

(2) $$g_{m}=\frac{\partial I_{D}}{\partial V_{G}}$$

**Figure 4:**
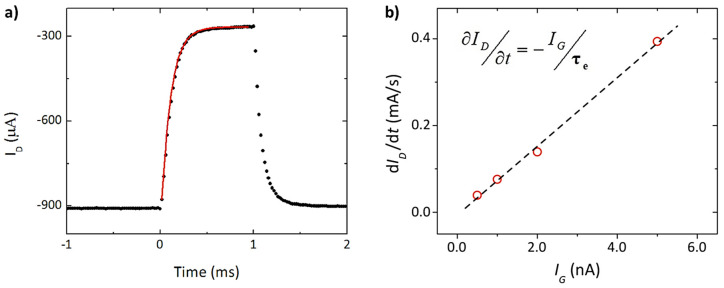
(**a**) Response time (τ_e_) of drain current in an OECT. (**b**) Dependence of temporal drain current (*I_D_*) on gate current (*I_G_*). The slope of the linear fitting brings the response time. Edited from Supp. Info. [10] (with permission from Springer Nature).

Another important characteristic of an OECT is the response time (τ) (Figure 4). The response time (τ), measures the time associated with the change in current on going from the “off state” to the “on state”. The time required for the movement of ions imposes an upper limit on the switching speed of the transistor, in analogy to the speed limit for a resistance–capacitor circuit (RC). The response time is limited by the movement of ionic species, which is traditionally considered as a long process (t = 1–100 µs).

Nevertheless, recent studies have shown that it is possible to measure the current at the drain with a short delay of only 20 µs after the application of the gate voltage. This response time is optimal for recording rapid biological signals [11].

**Figure 5:**
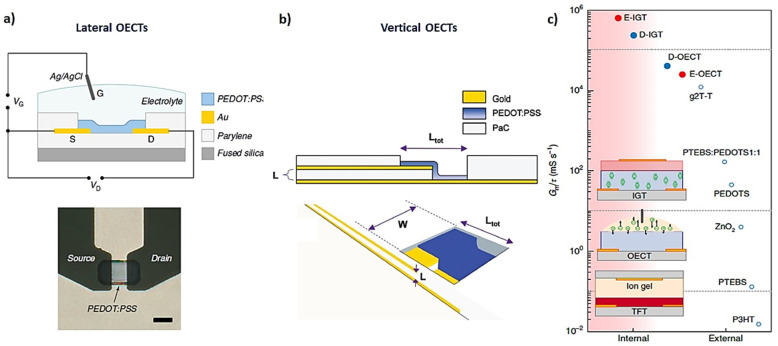
High-performance OECTs achieved by geometry design: (**a**) lateral OECT [10] (with permission from Springer Nature); (**b**) vertical geometry [14] (with permission from John Wiley and Sons, Inc.); (**c**) improvement of state-of-the art transistor performance by ion-gated electrochemical transistor (IGT) architecture (e-IGT and d-IGT; e = enhancement; d = depletion) [16,17] (with permission from Springer Nature).

An important benefit associated with the use of “soft” materials in the channel is the large range of device architectures that can be achieved (Figure 5), as well as different geometric factors and the possibility to integrate with different type of substrates, opening a large window of fabrication processes.

The most standardized fabrication method for OECTs used currently, consisting of a lithographic polymer-patterning process [12], was established by the Malliaras group. Briefly, the contacts, interconnects, and electrodes, generally made of gold, are deposited and patterned on glass substrates via lithographic methods. Then, two subsequent isolation and sacrificial layers based on Parylene-C films are adhered to the substrate. A soap solution, which works as an antiadhesive layer, is added between these layers. Afterward, windows are opened over the contacts and electrodes by photolithography and etching steps. The final polymer-based device is defined by deposition of the semiconductor material, generally via spin coating. This is followed by a physical peel-off step to remove the polymer and sacrificial layer from the regions where they are not required (Figure 5a,b).

In modeling the operating mechanism of OECTs, Bernards established an analytical expression that describes their electrical behavior in terms of ionic and electrical circuits and allows the fitting of their output characteristics [8]. Later, Rivnay presented a simplified expression (Equation (3)) that correlates the FoM, *g_m_*, with the geometrical OECT parameters when in the saturation regime for depletion-mode operation [13].

(3) $$g_{m}=\frac{W}{L}\cdot d\cdot µ\cdot C^{*}\cdot \left(V_{th}-V_{g}\right)$$

where channel dimensions *W* = width, *L* = length, and *d* = thickness, *µ* = hole mobility, C* = volumetric capacitance, and *V_th_* and *V_g_* = threshold and gate voltage, respectively.

This expression accelerated research in device design and enhancement to achieve devices with a higher transconductance, meaning higher amplification power. After the Planar devices design [10], devices with vertical geometries [14] were investigated in order to increase the *W*/*L* ratio and, in turn, achieve higher *g_m_*. Very recently, a new vertical architecture fabrication method was developed to decrease the channel length in an OECT device to the nano scale. These devices showed the highest transconductance (*g_m_* = 69 mS) published to date, opening a new path to the use of alternative non-photolithographical fabrication techniques [15]. This simple fabrication method proves that there is still much to explore for targeted applications, such as high-FoM OECTs in which the flexibility and stability of the devices are not detrimentally affected.

Moreover, Equation (3) emphasizes the importance of the volumetric capacitance (C*), which is the main difference between OECTs and their analogues, metal–oxide–semiconductor field-effect transistors (MOSFETs) and organic field-effect transistor (OFETs). In these devices, the amplification effect is due to a superficial field effect at the interface between the semiconductor material and the gate layer (Figure 5c). As discussed previously, in OECTs, this amplification stems from ions from the electrolyte penetrating into the bulk of the channel material, bringing a much higher amplification response.

Considering that all the other parameters are dependent on the geometry, architecture, and operating conditions (*W*, *L*, *d*, and *V_th_*, *V_G_*), the product of hole mobility and volumetric capacitance, *µ*·C*, has been recently established as an FoM to characterize the material used in the device channel. This FoM allows the comparison of different materials even if different device architectures are employed [9]. Inal et al. benchmarked different mixed-conductor materials used in OECTs and concluded that the ion mobility in the bulk of the polymer, which is related to the volumetric capacitance of the material, was the limiting factor to achieve high-FoM materials. This is due to its influence on the device response time required for high-performance OECTs.

Recently, Spyropoulos and colleagues developed a new best performer design of OECT, the internal ion-gated electrochemical transistor (IGT) [16,17]. In these devices, the semiconducting channel material (composite PEDOT:PSS/polyethylenimine (PEI)) is intimately in contact with hydrated ion reservoirs (e.g., d-sorbitol in the presence of ion membranes using materials such as chitosan) which reduces the time taken for ions to move into the channel. This increases the operating speed of the transistor due to the faster dedoping process (due to the redox state change), and the response time (τ) is then a hole-mobility-limited process (fast, 0.1–10 cm^2^·V^−1^·s^−1^) rather than an ion-mobility-limited process (slow, 10^−2^–10^−4^ cm^2^·V^−1^·s^−1^) as in the case of standard OECTs and EGOFETs (electrolyte-gated OFETs) [18,19,20,21].

The authors of this study developed both depletion- and enhancement-mode devices (d-IGT and e-IGT) where the redox state of the semiconductor material is controlled due to the self-doping capability of the composite channel material, with no requirement for an external ionic source (Figure 5c). This work addressed one of the major bottlenecks which OECTs had to overcome in order to be suitable for demanding applications such as integrated organic circuits for bioelectronics, where the high performance of both modes of operation allows a large range of targetable and miniaturized designs for particular applications.

In order to adapt OECT fabrication methods to meet the requirements of the large range of applications where their amplification power and versatility could be exploited, a great effort has focused on exploring different printing techniques for the fabrication of high-performance OECTs. For a thorough overview of these different printing techniques, a recent review can be consulted [22]. These approaches are primarily aimed at flexible and wearable applications. Finally, it is noteworthy that ionic liquids or gels have also been used as electrolytes to develop OECTs, opening new pathways for enzymatic electrochemical sensors [23].

## 3. Organic Semiconductor Materials for OECT

An organic semiconductor material (OSM) is a type of carbon-based material that exhibits interesting optical and electronic properties for use in a myriad of applications. These materials have attracted increasing research interest over the last few decades, mainly due to industrial interest in the development of applications such as flexible and portable solar cells or organic photovoltaics (OPVs) [24]. This increased interest enabled the achievement of mature technologies that can be found in our daily lives, for example, in smartphones and colorful screens (organic light-emitting diodes, OLEDs) [25], as well as increasing development of switches (transistors) used in biosensors [26]. One important advantage of these organic materials is that their molecular properties can be tuned by chemical design and adapted to suit the targeted application.

The first organic conductive material, polyaniline, was described in 1862 by Henry Letheby [27]. Almost a century passed by until this research field gained the general interest of the scientific community, and it was only from 1970 onward that efforts focused on the development of organic electronics thanks to novel polymers similar to polyaniline [28,29].

The OSM structure is based on the *sp*^2^ configuration of carbon atoms instead of silicon atoms traditionally used in electronics. This family of materials is classified into two groups on the basis of the weight, namely, π-conjugated polymers and small molecules. The common property is that they are π-conjugated systems. This means that they are characterized by the presence of conjugated bonds forming a delocalized π-electron cloud. This conjugation is the origin of one of their characteristic properties, their electrical conductivity, which falls between that of a conductor (such as metals) and an insulator (such as glass).

Although, in most of cases, these materials have a relatively low electrical conductivity, their conductivity can be modified by the controlled introduction of impurities (additives) into the crystal structure. This process, known as doping, brings them to a doped state, improving the transport of electrical charge. In many examples, this transition between the two states (undoped and doped) is reversible because it occurs through a redox mechanism. Doping can be negative (n-doping) or positive (p-doping) depending on the nature (charge) of the dopant used. Moreover, the transition between these two states can be induced by ion exchange between the semiconductor material and an electrolyte which is in contact with it [30,31].

As stated before, generally, organic conductive polymers present much lower conductivity than their inorganic alternatives; however, these materials present other advantages. Their high processability (possibility to deposit them on surfaces such as screens by printing) and their specific mechanical properties (i.e., flexibility) have allowed the continuous development and application of new materials.

**Figure 6:**
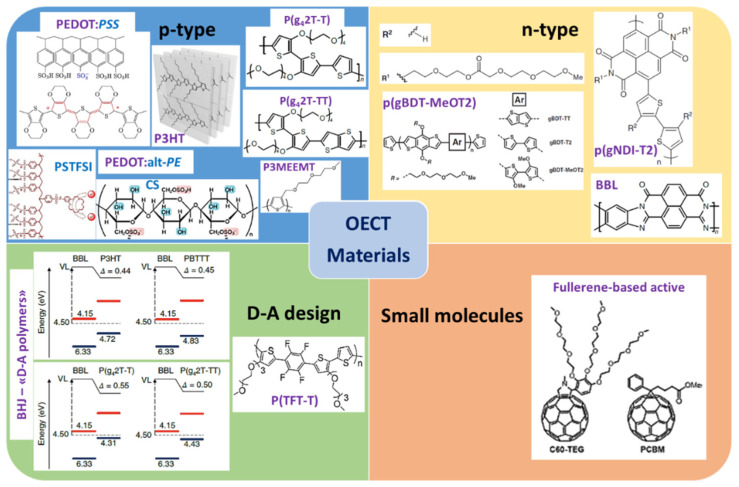
Summary of representative OECT materials. Chemical structures of: *p-type materials* such as PEDOT derivatives including the most used material PEDOT:PSS [30,31,33,38,51,59,64,66] and other thiophene derivatives [32,37,70,71,78,80]; *n-type materials* based on thiophene-based building block modifications or ladder polymers [87,88,89,90,91]. *Donor–acceptor (D–A) strategy* such as integration of rigidizer fluoroaromatic rings or heterojunctions based on the combination of D–A polymers [84,85]. *Small molecules* as active channel materials [94]. (With permission from John Wiley and Sons, American Chemical Society, and Springer Nature).

The most important semiconductor materials used as the channel in OECT devices have traditionally been p-type, although recently McCulloch and coworkers have shown that the use of a n-type polymer can also provide OECT behavior [32]. This section gives an overview of the materials explored in OECT devices in recent years. A graphical summary is provided at the end of the section (Figure 6).

### 3.1. Conducting Polymers—p-Type

The most-commonly used channel material in OECT devices is based on the p-type material PEDOT and its derivatives [33]. However, other p-type materials have been developed, initially for OPV technologies [34], and the improvement of their chemical and physical properties has triggered high interest in the scientific community [35,36], leading to their recent integration in OECT devices [37]. This section gives an overview of p-type materials used in OECT devices in recent years (Figure 6, blue panel).

#### 3.1.1. PEDOT and Some Derivatives

One of the most important materials exploited in the organic electronics field is poly(3,4-ehtylenedioxythiopene) (PEDOT). This polymer has gained major attention due to its numerous advantages for this field, such as good charge transfer, high chemical and electrochemical stability, and simple film deposition [30,31,38]. In this section, a brief overview of the main particularities of PEDOT and its derivative materials is presented. They are the most used materials in bioelectronics, due in particular to their “soft” nature, a property that makes them good candidates for interfacing with biological tissues [39].

There are three main different mechanisms of polymerization of PEDOT, and they were recently reviewed by Donahue and collaborators who shed light on the influence of the processing conditions used on the final material properties [40]. Briefly, we can identify two broad methods: electrochemical polymerization (EP) and chemical oxidative polymerization (COP). Within COP, we can distinguish a subcategory, the vapor polymerization methods, i.e., vapor-phase polymerization (VPP) and chemical vapor deposition (CVD).

Electrochemical polymerization: An applied electrical voltage oxidizes the monomer in the electropolymerized PEDOT. The film morphology and performance depend strongly on the dopant anion and the parameters used during the deposition (substrate, electrochemical method used, potential, solvent, scan rate, etc.). The most used counterions or dopants are low-molecular-weight anions (i.e., PF_6_^−^, ClO_4_^−^, or tosylate (Tos)^−^), but polymeric counterions (i.e., PSS or *S*-phenylalanine (*S*-Phe)) and biopolymers (i.e., heparin, dextran, or hyaluronic acid) have also been used. The main limitation of this method is that a conductive substrate is needed. Delamination can also cause problems for thick films, where substrate-dependent adhesion issues occur [41].

Chemical oxidative polymerization: This is induced by the chemical oxidation of the monomer catalyzed by oxidizing agents such as iron derivatives (i.e., FeCl_3_ or Fe(Tos)_3_) or persulfate salts (i.e., (NH_4_)_2_S_2_O_8_). In this method, the substrate variability is larger due to the from-solution film processability. Moreover, different additives can be introduced in the reactor or in the formulation vial (cosolvents, cross-linkers, surfactants, etc.) to obtain highly conductive, transparent films with good mechanical properties [33,42,43,44].

Vapor polymerization methods: These can be considered as a subcategory of chemical oxidative polymerization because of the requirement for an oxidizing agent. Different methods are based on different approaches to the application of the oxidizing agent. On the one hand, in the case of CVD, the oxidant is deposited as a vapor in the reaction chamber. On the other hand, in the case of VPP, the monomer is deposited after previous deposition of the oxidizing agent on top of the substrate. Careful control over the process parameters (rate flow control, temperature, etc.) is necessary as these influence the crystal growth and the film morphology. These, in turn, influence the π–π stacking interactions, determining film properties such as conductivity and rigidity [45,46,47,48,49].

The most used material over the last decade has been the previously mentioned PEDOT:PSS. This is a complex mixture between the semiconducting polymer PEDOT and a polyelectrolyte containing sulfonic groups, PSS. The PSS stabilizes the quinoid conformation of PEDOT (Figure 6, blue panel). The electrochemical polymerization of EDOT from an aqueous PSS solution was published for the first time by the Yamato group [50]. In its oxidized state, PEDOT:PSS is conductive due to the presence of charge carriers along the conjugated chain [51,52,53]. Due to this property, as well as its transparency and commercial availability as a water dispersion, this material has long been used as a hole-selective layer in OPVs. This layer allows the rugosity of the ITO to be reduced and blocks the flow of electrons. Unfortunately, its hygroscopic and acidic nature brings chemical instability of the active layer and degradation of the solar cells. These reasons and a desire to extend this material to new applications motivated the organic electronics community to find alternative materials to overcome these drawbacks.

In one example, PEDOT:biomolecule dispersions were designed, changing the electrical conductivity, as well as the mechanical properties, of these hybrid systems [54,55,56,57,58]. Remarkably, Horikawa et al. achieved high electrical conductivity with cellulose-stabilized PEDOT films (Figure 6, blue panel). This was attributed to a greater proportion of this quinoid crystal structure in this blend in comparison to PEDOT:PSS when the optimized degree of sulfate groups in the cellulose was used [59]. More complex mixtures were created by blending these PEDOT:biomolecule systems with a third nonconductive element to enhance their properties. A good example of this is conducting polymer hydrogels in which the conductive polymer is embedded in a hydrophilic cross-linked polymer matrix. The resultant material combines the electroactivity of the PEDOT with the mechanical properties of the nonconductive polymer [60,61,62,63].

Interesting synthetic work sought alternatives to this commercial material, substituting the PSS dopant with alternative polyelectrolytes, such as poly(styrene sulfonyl(trifluoromethylsulfonyl)imide) (PSTFSI) and obtaining similar PEDOT-based aqueous dispersions [64]. These alternative materials can be used to extend printability to large-area substrates, and they have found a place in applications requiring flexible transparent electrodes [65].

Recently, it has been shown that targeted properties can be achieved by chemically tuning the polyelectrolyte structure (Figure 6, blue panel). In this example, K^+^ monovalent cation selectivity was observed in the derivative aqueous PEDOT inks when the appropriate cation scavenger was integrated into the polyanion structure [66]. This property, coupled with the high capacitance and biocompatibility of these materials, can be exploited in ion-sensitive applications using OECTs as sensors.

Nevertheless, PEDOT:PSS remains the most used [40] and modeled [67,68,69] standard material in the OECT community, mainly due to its commercial availability and easy processability, as well as the vast knowledge accumulated on the tunability of its properties. This material has enabled the realization of high-performance devices, but it is generally limited to depletion-mode operation which inherently consumes more power than a transistor operating in accumulation mode.

#### 3.1.2. Other Thiophene Derivatives

In the exploration of alternative materials to PEDOT:PSS toward the enhancement of device performance and functionality in the field of OECTs, researchers sought inspiration and opened the toolbox for OPVs and OLEDs, as these were the research focus of the organic electronics community for many years. Thus, the second family of materials most commonly studied for use in OECTs constitutes the thiophene derivatives. This is due to the development of robust methods for the synthesis of regioregular polymers with controlled molar mass and end-chain functionalization [70,71,72].

- P3HT Family: Integration of Functions

Thiophene is an organosulfured heterocycle whose aromatic nature is the origin of a large number of substitution reactions. Its polymers, polythiophenes, form one of the most environmentally, chemically, and thermally stable conducting polymers in the doped and dedoped states which has led to its extensive use in organic electronic devices. Its low solubility in common solvents, the formation of undesired side products (due to α and β couplings during its polymerization), and its high oxidation potential have a deleterious effect on its conductive properties [73]. Thus, modification of this precursor was a cornerstone topic in organic electronics in the 2000s, achieving, thanks to intense research efforts, representative materials such as PEDOT and its derivatives already discussed [33,36,51,52,53].

Important approaches to overcoming the drawbacks of polythiophenes have included the introduction of long alkyl side-chains onto the aromatic unit and the development of a controlled polymerization method [70,71]. The most representative polymer of this family, the regioregular poly(3-hexylthiophene-2,5-diyl) (P3HT), has seen extensive use in OPVs [74] and OFETs [75] (Figure 6, blue panel). The easily processability of this polymer in comparison with nonfunctionalized polythiophenes, as demonstrated in these previous studies, highlighted this polymer as an interesting candidate for OECTs. It should be noted, however, the electroactive window of P3HT polymer, in the presence of organic or ionic liquid electrolytes, is in the high-voltage range [76]. This is incompatible with biological conditions, the main field of application for OECTs. This was the driving force for the use of other thiophene derivatives and the development of new materials.

Remarkable efforts have focused on obtaining P3HT derivatives for use as high-performance mixed conductors. Stingelin et al. studied random copolymers of hydroxyl-functionalized P3HT showing that addition of hydrophilic lateral chains to the polymer backbone allowed these materials to be employed in aqueous electrolyte at low applied voltages [77]. This work demonstrated the key role that morphology and hydrophilicity play in mixed-conductor performance.

The highlighting by Malliaras et al. of the fundamental properties required for the adequate design of materials for OECTs, i.e., a high electrical mobility and high volumetric capacitance [9], has boosted the interest in new design strategies and the elucidation of material design vs. device operation mechanisms.

Another example of continuous effort on the synthetic development and characterization of thiophene derivatives and hybrid materials is the work of Luscombe and coworkers. Recent work showed excellent figures of merit for accumulation-mode OECTs based on a modified polythiophene polymer integrating side chains made of ethylene-glycol (Figure 6, blue panel), poly(3-{[2-(2-methoxyethoxy)ethoxy]methyl}thiophene-2,5-diyl) (P3MEEMT) [78]. The authors called attention to the influence of the anion nature on the OECT performance. Some examples are the dependence of threshold voltage (V_th_) on the anion used, as well as anion-dependent charge injection (comparing the smaller charge density of PF_6_^−^ vs. Cl^−^). This has a strong impact on the polymer hydration during the doping process, resulting in an important packing-density effect. This causes faster kinetics in P3MEEMT in comparison to reference P3HT. An in-depth study of the doping mechanism was presented this year, where the authors again pointed out the importance of understanding the charge compensation mechanism to improve the design of mixed conductors for organic electronics applications [37].

Nielsen and coworkers synthesized different thiophene-derivative precursors, such as triethylene glycol (TEG), well known for its electron-rich conjugate system incorporating plasticizer side-chains, to optimize ionic and electrical transport in the entire volume of the polymer film, thereby improving the performance of accumulation-mode OECTs [32]. Polymerization and copolymerization of these building blocks allowed them to achieve a library of materials with a range of backbone curvatures and ionization potentials between 4.4 and 4.9 eV (Figure 6, blue panel). From this family of materials, the best performing was p(g2T-T) with a high transconductance (*g_m_*^max^ = 20 mS), an on/off ratio = 10^5^, low-voltage operation (0 V), fast response time (ms regime), and high stability. In a further step, in a comparison of alcoxylated (a) vs. glycolated (g) analogues of a similar precursor 2T-TT (one fused thiophene unit was added to the building block with respect to g2T-T), the achievement of high performance with steep switching, little hysteresis, and excellent stability in aqueous media was shown when the glycolated version, p(g2T-TT), was used. In the opposite case, the alcoxylated version showed a mix of interfacial and bulk doping [79]. Recently, Cendra et al. used spectroelectrochemical techniques to deeply investigate changes in the microstructure and morphology during operating conditions which shed light on the anion dependence of the polymer-hydration state [80].

These works show the complex interplay between mixed conduction, chemical design, and operating conditions, as well as the important role that kinetics plays in the material doping, affecting the device operation in a significant way. This highlights the fact that material design and understanding of mechanisms is required, and that the systematic adoption of the learnt strategies from OFET technology is not sufficient.

- Donor–Acceptor (D–A) Design

Following the previous approach to chemical design, but this time focusing on the enhancement of charge-carrier mobility (Figure 6, green panel), one can notice the work done to reduce the structural and energetic disorder by rigidifying the polymer backbone [81,82,83]. In the design of materials for OECTs, Parr and coworkers recently explored these strategies to obtain donor–acceptor polymers based on tetrafluorophenilenes and triethylene glycol-functionalized aromatic rings, obtaining a hysteresis-free OECT with remarkable performance (µC* = 10.0 F·cm^−1^·V^−1^·s^−1^) [84]. 

Recently, Xu and coworkers studied all-polymer donor–acceptor heterojunctions [85]. Instead of just integrating donor and acceptor moieties into the polymer chain, they combined polymers with low ionization energy with high-electron-affinity polymers. This brought about a considerable increase in the polymer interface conductivity due to the parallel electron and hole distribution, which allowed excellent spontaneous ground state electron transfer (GSET) of the D–A interface (Figure 6, green panel). Moreover, bulk heterojunctions obtained with these materials possessed high stability opening the way to promising wearable applications.

### 3.2. Conducting Polymers—n-Type

Materials that overcome some of the limitations associated with p-type polymers have started to emerge. Taking the case of PEDOT derivatives, their device operation is mainly restricted to depletion mode, presenting little difference between the on/off states during switching at low bias voltages. However, a high signal-to-noise (S/N) ratio is required for signal amplification in some demanding biological applications [86]. Thus, after the first demonstration of a high-performance OECT operating in accumulation mode by McCulloch and coworkers [32], the pathway was opened to the development of new n-type materials and the elucidation of their structure–property relations for OECT applications. This section gives an overview of the n-type materials explored in OECT devices in recent years (Figure 6, yellow panel).

In a similar approach to the modification of thiophene-based building blocks to obtain materials such as p(g2T-T), Giovannitti continued exploring the modification of bithiophene precursors through the incorporation of a naphthalene derivative, NDI, to obtain materials with hybridized energy levels [87]. The authors fabricated ambipolar transistors with p(gNDI-T2) showing p-and n-doping in aqueous solution, as well as good stability after 2 h of operation. However, due to the highly localized charge carriers on the chain due to the donor–acceptor character, the OECT performances obtained were limited in comparison with more classical p-type OECTs in accumulation mode. Another example of the retrosynthetic approach followed by Giovannitti addressed the instability of alkoxy-benzo[1,2-b:4,5-b′]dithiophene (alkoxy-BDT) polymers by changing the building-block structure through co-monomerizing the BDT unit with an electron-rich moiety of 3,3-dimethoxy-2,2′-bithiophene (MeOT2) [88]. This co-monomer allowed the charge stabilization to be increased during electrochemical oxidation, as confirmed by the highly stable OECTs obtained (Figure 6, yellow panel).

Sun et al. explored the use of the semicrystalline ladder-chain polymer poly(benzimidazobenzophenanthroline) (BBL) as the active material in OECTs fabricated by simple spray-coating. These devices showed high stability after storage in air for months and under aqueous device operation for 1 h under gate pulses [89]. These devices represent the best-performance n-type OECTs reported to date (*g_m_* = 9.7 mS, C* = 900 F·cm^−3^). This was attributed to the good intramolecular charge transfer and the high n-dopability due to the presence of redox-active sites in the poly(benzimidazobenzophenanthroline) polymer [90,91]. Nevertheless, the response time is still limited due to slow ion diffusion into the polymer layer, suggesting that future efforts should be focused on improvements by material engineering.

### 3.3. Small Molecules

Most of the OECTs developed until now have been based on conjugated polymers. Nevertheless, semiconducting small molecules are of great interest for OECTs due to their higher electronic mobility (20–40 cm^2^·V^−1^·s^−1^) in comparison with their polymeric counterparts [92]. In this section a brief overview of the recent use of small molecules as active layers in OECTs is given (Figure 6, orange panel).

Even though many n-type small molecules, mainly acceptors, are well established in OPVs [93], these materials are just emerging in the OECT field. Bischak et al. were the first to use a fullerene derivative to form the channel layer in OECTs [94]. Exploring techniques learnt from conjugated polymers, they showed that 2-(2,3,4-tris(methoxtriglycol)phenyl)[60]fulleropyrrolidine, C60-TEG, presented excellent properties as the n-type channel material in these devices, obtaining excellent figures of merit (µC* = 7.0 ± 2.0 F·cm^−1^·V^−1^·s^−1^) compared to recent conjugated polymers research. This lays the groundwork for the use of processable thin films based on new small molecules which present good ion transport, as well as high electronic charge transfer.

In conclusion, the key role of chemical design is confirmed through analysis of OECT performance and physical–chemical characterization of the materials. For instance, the most structurally ordered polymers, which present better charge-transport properties, show better electrochemical performance as OECT devices. Complementarily, small-molecule semiconductors are promising materials due to their higher electronic mobility, and we foresee the inclusion of these materials in high-performance OECTs.

## 4. Applications

OECT-based sensors have been the subject of increasing research interest over the last few decades, mainly due to the great demand for high-performance chemical and biological sensors in diverse areas ranging from food-safety tests to agricultural and environmental monitoring, as well as medical and healthcare or security applications [95].

It is helpful to consider the key difference between a chemical and a biological sensor. In a chemical sensor, the measurement of the chemical information of a studied system (i.e., the analyte concentration) is performed though the correlation of a physical property with a useful analytical signal (e.g., absorbance). In a biological sensor, the analyte detected has a biological origin (i.e., enzymes, antibodies, DNA, proteins, micro-organisms, etc.) [96].

**Figure 7:**
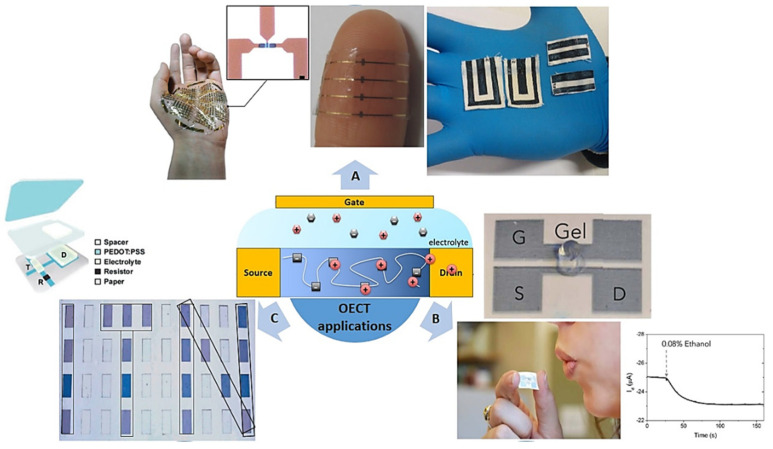
Flexible and wearable devices based on OECTs. (**A**) Flexible printed sensors for wearable and e-skin applications [17,137] (with permission from Springer Nature). (**B**) Paper-printed alcohol sensor [138] (with permission from Springer Nature). (**C**) OECT-based logic circuit: the message “ITN” is projected by electro-chromic properties [149] (with permission from John Wiley and Sons).

OECT devices are promising for “translation” of small changes in ion concentration to large changes in electrical current, a property which is really exploited in bioelectronics applications. These devices have started to emerge as components in logic circuit technology and internet of things (IoT) applications. Finally, if this technology is to be more than a scientific curiosity and be adopted for use in future portable and wearable applications, some properties such as lightweight and flexibility, stability under normal operating conditions, low-power operation, and continuous recording, must be achieved. Here, we present some examples of the increasing relevance of OECTs in this range of applications (Figure 7).

### 4.1. Biology and Sensing

Bioelectronics is a field which has seen a massive increase in research investment in recent years [39,97]. Today, this includes a broad range of applications going from medical devices to sensors for the detection of molecules in the context of environmental protection. On the medical device side, numerous patients have seen an improvement in their quality of life thanks to devices such as cardiac pacemakers, cochlear and retinal implants, and glucose sensors. Being now part of our daily life, bioelectronics have received notable attention in the media, due to either science fiction or real-life research advances [39,98].

The general organic electronics field has received great attention for bio-applications because of its material versatility, whereby their mechanical, chemical, and physical properties can be precisely controlled and modified to suit specific applications [99]. In particular, two properties have promoted the rise of the organic bioelectronics field: the biocompatibility of these “soft” materials in comparison to their inorganic counterparts and their mixed conduction ability (ion and electronic mobility). The reported performance of OECTs has considerably improved in recent times, and they have already supplanted the biosensing capabilities of alternative devices (i.e., conductive-polymer-coated electrodes or thin-film transistors) [10,79,92]. Another reason for the increasing research interest in OECTs is due to the intrinsic limitations of alternative methods. For example, high-spatiotemporal-resolution optical methods are hindered by potential heating of the cells’ environment coming from photostimulation. In the case of genetic probes, ethical and safety issues may arise, and accurate massive modification is still not possible even if these fields advance in an impressive way [100,101].

The first examples of the use of “classical” conducting polymers, i.e., polypyrrole (Ppy), presenting a study of a bio-inorganic interface came from Langer’s group [102,103]. In the analysis of these interactions, they realized that the conducting polymer state (reduced/more neutral or oxidized) has an important influence on the cells’ properties, such as their ability to anchor to the electrode surface or their growth and morphology. A further step led to the use of these conducting polymers as the active layer in sensing devices. A representative example is given by the performance enhancement of analogue OFETs demonstrated by the Torsi group’s work. Some examples are the discrimination of chiral analytes or the channel bio-immobilization demonstrated on these devices [104]. Nevertheless, due to the superficial active area of these devices in comparison to the volumetric one of an OECT, a higher amplification power is obtained from the latter. Finally, the improvement of water stability of OFET sensors is a must for medical and biological applications.

Recently, efficient detection of lactate, an important cellular metabolite, has been reported with n-type OECTs [105]. The fast exchange between the different redox states of the channel material comes from the electrons generated by the enzymatic reaction. Thus, this is an example of sensitive and selective metabolite sensors that could be obtained with the high signal-amplification power of OECTs, exceeding traditional amperometric sensors. Moreover, due to this amplification property, these devices appear to be an interesting alternative to microelectrode arrays (MEAs) for electrophysiological recordings. In fact, Hsing and coworkers demonstrated their efficiency in the study of cardiomyocytes, monitoring cardiac spikes with an average S/N record of 4–10 [106]. This first demonstration of long-term noninvasive recording is encouraging and motivates the assessment of OECTs for drug screening and fundamental in vitro research applications. Nevertheless, various outstanding issues surrounding OECTs, such as their technicity simplification (i.e., coupling recordings set up with a unit for in situ analysis treatment), stability (to the biological, chemical, and physical treatments in the device fabrication and in operation conditions), or even their commercial viability, will need to be addressed.

A further step was the application of OECTs for in vivo electrophysiological recordings. In 2013, Khodagholy and coworkers showed that engineering of ultrathin OECTs allow low-amplitude brain activity to be recorded with unprecedented S/N due to the local amplification in comparison with metallic electrodes [107]. This interesting work attracted a lot of attention due to the possibilities that this approach could bring for healthcare and diagnostic applications, as well as brain–machine interface development.

Nonetheless, for real applicability of OECT devices in challenging applications, such as clinical diagnostic equipment, the limit of detection (LOD) and the specific window of concentration regime that is possible to sense (sensibility) are of extreme importance. These values are very diverse depending on the targeted analyte and the body fluids and/or organs of interest; however, recent studies have shown that the amplification power and design of OECTs can achieve superior analytical performance (LODs, sensitivity, selectivity, reusability of the device, etc.) compared with alternative electrochemical and analytical methods. In a recent example, improvement of the LOD for dopamine with an aptamer–OECT-based sensor was demonstrated where the detection limits decreased to the femtomolar range (in comparison with micromolar concentration limits of an analogue amperometric aptamer sensor). Thus, detection of dopamine in complex media (where the presence of other species with similar oxidation potentials, such as ascorbic acid and uric acids, could interfere with the specific dopamine interaction) can be envisaged with OECT-based sensors [108].

To demonstrate the large range of possibilities afforded by using OECTs as amplifying sensors, a brief summary with representative materials and sensing analytical parameters is presented in Table 1.

### 4.2. Future in Wearable Devices

Lightweight, wearable, and low-power OLEDs have appeared in on-skin medical applications such as cancer treatment [117] or muscle-contraction sensors for robotics [118]. All-organic flexible devices offer an alternative to traditional rigid oximetry sensors [119]. Today, we live in a world demanding excellent in situ and noninvasive sensors, a market where OECTs present intriguing prospects. From the mentioned examples, considering the requirement for contact with skin or other biological systems, it is necessary to achieve specific material properties, as well as device architectures, compatible with the mechanical stress and long-term use to which these devices will be subjected [3,40,120].

Nevertheless, as is clear from the precedent examples, many of the technological bottlenecks in the production of flexible devices have already been solved for other organic electronic devices [121]. This could be of significant help to the analogous development and application of OECTs. Here, we present an overview of the advancement of research on flexible wearable OECTs (Figure 7). Most flexible organic printable technologies were developed initially for electronic papers [122], printed electronic circuits [123,124,125], and more recently for e-skin applications [126,127,128]. In these times, emerging biomedical devices in service to society have started to change the way we sense, record, or analyze human health parameters [129].

For these kinds of applications, flexibility and stretchability are the major requirements, and devices that can be placed anywhere on the body are sought after. It is possible to already find innovative designs in biomedical devices. For example, some have similarity to tattoos, adhesives adhered to the skin or soft conductive gels creating high-quality electrical–skin interfaces [130,131,132,133]. In this type of electrophysiological sensor, the pattern of the biosignals (i.e., electrocardiograms (ECG) for the cardiac cycle, electrooculography (EOG) for eye movement, or electroencephalography (EEG) for neural rhythms) is used to detect a particular pathological state [134,135]. For this reason, a high S/N ratio is required in the recordings. This is generally obtained by reducing the sensor impedance at the interface with the skin by minimizing the sensor size or combining with a conductive polymer as already mentioned. This could be improved through the use of an OECT if a high ion mobility in the bulk of the polymer film was guaranteed.

Recently, the first examples where printed OECTs were exploited in medical diagnostics and monitoring have been reported, e.g., for electrocardiography [136]. In this example, the authors patterned OECTs on a bioresorbable three-dimensional (3D) scaffold and showed excellent electrocardiogram recordings comparable to standard faradaic electrodes.

Targeting wearable and portable applications, another challenge is the printing of devices on tissues. The first steps to this were shown by Gualandi et al. who developed a textile-embedded noninvasive OECT sensor by screen printing in a single-step process [137]. The authors distinguished different biomarkers (adrenaline, dopamine, and ascorbic acid) in biological fluids and artificial sweat showing similar sensing capabilities to standard flat OECTs. Moreover, these sensors showed low-voltage operation, as well as a high stability to repetitive hand-washing cycles, making them very promising wearable biosensors. In a similar approach, this time targeting in situ sensing applications, Bihar and coworkers presented a printed OECT-based alcohol sensor on paper, an inexpensive, disposable, and biodegradable substrate [138]. This proof of concept is a good example of simple and robust sensors that could be integrated in portable devices and allow, through IoT technology, different security applications.

One of the most challenging applications to develop in stretchable bioelectronics is implantable sensors. This is due to the fact that, for surgical operations, requirements of biocompatibility, stability, and long-term function are more emphasized and stringent [139,140]. For this reason, soft materials including conducting polymers could be preferable as they would reduce tissue damage in comparison to their rigid inorganic counterparts [141,142].

As a less invasive option, we can distinguish the use of OECTs on surface arrays showing the possibility to detect small-amplitude and local biosignals with a higher S/N ratio in comparison to flexible electrodes [107]. A step further has been their use in implantable in-depth probes, which could shorten the way toward clinical assays [143,144,145].

Williamson and coworkers addressed the issue of brain-tissue damage by embedding the OECTs in a parylene isolator layer to reduce the probe invasiveness [146]. They showed specific local neural stimulation in the brain hippocampus when current pulses were injected from the device channel. This overcame limitations of more traditional electrode probes which need thicker encapsulation, thus losing the flexibility of the probe. Moreover, it demonstrated the possibility of using OECTs for in vivo brain research and clinical studies.

In 2020, Cea and coworkers showed excellent long-term in-depth probe recording in the brain cortex with a new OECT design, the ion-gated electrochemical transistor [17]. The high performance of these devices stems from the ingenious integration of a hydrated ion reservoir intimately with the polymer composite channel (PEDOT:PSS/PEI), reducing the response time of the transistor due to the rapid transit of ions from this reservoir to the semiconducting polymer [16]. This design allows unprecedented performance and long-term stability to be achieved under electrophysiological recording conditions, such as those used for EMGs and ECGs, detecting neurophysiological signals. This lays the foundation to use these e-skin devices as noninvasive electrophysiological transducers. Furthermore, more impressively, surface and in-depth encephalography were also performed with implantable probes in freely moving animals. From the cortical surface local field potentials (LFPs), brain activity was detected during 2 weeks of recordings, showing the robust device’s stability and biocompatibility. Moreover, in-depth probes were inserted in deep cortical layers, allowing recording and processing of in situ characteristic high-frequency spike activity (action potentials, APs) from individual neurons in vivo. This is a striking example of the potential of OECT devices, where design optimization enables progress toward chronically implanted bioelectronic interfaces.

A big difference between the current settled electronics technology and emerging organic electronics is not based on device conception (we can find OLEDs in front of LEDs, OECTs vs. TFTs, etc.), but instead on complementary organic circuit fabrication. Now that stable and well-performing n-type OECTs have appeared, the use of organic electrochemical transistors (p- and n-type) could allow the achievement of circuits with faster switching speeds, as well as more stable operation and lower power dissipation [147].

The first example of organic logic circuits was presented by McCoy and coworkers. Using a microassembly of OECTs, they obtained the first organic crossover distortion-free amplifier [148]. Later, Andersson showed a novel concept for electronic papers presenting an electrochromic pixel matrix display based on PEDOT:PSS electrochemical transistors [149]. This device was able to display clear text messages (Figure 7c). Berggren and colleagues advanced further by designing organic-based logic circuits for complementary circuits, which were until then based on solid-state OFETs [150]. In this work, the authors designed an OECT-based circuit and showed its operation as an oscillator and NAND and NOR logic gates. Later, the same group showed the first totally electrochemical-based complementary inverters. Sun et al. combined p-type (P3CPT) and stable n-type (BBL) OECTs, in aqueous and long-term air storage, achieving high gain at low supply voltages. This is of great interest for future applications in biosignal amplification [89].

Having in mind the complexity of metabolite detection discussed previously, Braedlein and coworkers used a Wheatstone design for an in vitro OECT-based circuit to detect, within a few seconds, ex vivo lactate in complex cell culture media from stimulated tumor cells [151]. This sensor design allowed any interference in the signal to be removed, by comparison of both transistors’ outputs, and showcased the applicability of this type of circuit in clinical control protocols.

Recently, Khodagholy and coworkers reported the best-performing integrated circuits based on IGTs, already mentioned before [17]. This work overcame an important bottleneck in bioelectronics, by addressing the requirement for not only in situ acquisition of the biosignals, but also the real-time processing of these signals. The integrated circuit built consisted of a nonlinear rectification system combining IGT transistors working in depletion and enhancement mode, d-IGT and e-IGT. The combination of its low and high off-currents avoids an additional channel-patterning step because of the negligible electrical current between adjacent transistors. This simplifies the fabrication process and guarantees its scalability, also showing minimal gate-current leakage. This design with tunable properties resulted in soft, biocompatible, and implantable processing units, as well as the achievement of a clean or “filtered” signal by the suppression of low-amplitude nonspecific events. This shows the great potential of this type of transistor to obtain in situ sensors and processing units which could give birth to a new generation of chronically implantable bioelectronic devices useful for research and clinical studies of important electrogenic deficient pathologies, such as epilepsy, Parkinson’s, and diabetes [134,135].

## 5. Conclusions and Perspectives

The common requirement in a large range of emerging technologies is excellent ion transport. Throughout this paper, we discussed several applications of OECTs, going from bioelectronics to logic circuits. OECTs gather all the assets needed to achieve the mixed (ion and electron) conduction needed in these applications: from detection and processing of small biosignals with unprecedented amplification power to their integration in flexible wearable substrates.

In summary, the volumetric effect in the channel material gives a bigger amplification in OECTs compared to their analogues. OECTs work within low voltage ranges compatible with biological media, and the design flexibility allows a broad spectrum of applications to be targeted (i.e., rigid or flexible applications, different geometries, immobilization of receptors to targeted analytes, variation of channel materials, etc.). This makes it possible to develop disposable biosensors with high limits of detection, sensitivity, and selectivity.

This continuous and fast performance enhancement has resulted in exciting proof-of-concept devices and is expected to lead to promising future devices in service to society, from healthcare and diagnostics to logistics applications. However, there is still plenty of room to improve and better understand these technologies. For example, simulation and theoretical calculations could be utilized to better comprehend how material structure–property relationships influence device operation. Furthermore, the development of not only “proof-of-concept” devices, but also more in-depth studies regarding standardizing, long-term stability, and reproducibility could expedite the development of the first commercial devices and long-term clinical applications in bioelectronics.

## Figures and Tables

**Table 1:** Summary of analytical performances of different OECT-based sensors.

Material	Ref.	Max *g_m_* (mS)	Max *I_D_* (mA)	Δ*V_g_*^eff^ (mV·dec^−1^)	Device Sensitivity	Application	Analyte	LOD Sensitivity	Selectivity
PEDOT:PSS + SplitAptamers (Gate)	[108]	4	2.5	-	-	Neurotransmitter sensing	DA, AA, UA, GABA	0.5 × 10^−15^ M; 10^−15^–10^−9^ M	Dopamine (DA)
PEDOT:PSS + Glycan + PDCNT (Gate)	[109]	0.4	0.275	11.4	-	Cancer cells detection	Mannose	10 cells/uL; 10^4^–10^5^ cells/uL (sat)	MCF-7
PEDOT:PSS + m-antiHER2 (Gate)	[110,111]	-	-	-	-	Cancer protein biomarkers detection	HER2	10^−16^ M; 10^−7^–10^−14^ M	HER2 biomarker (10 cells/uL)
P3HT	[112] *	-	0.012 *	62 *	0.25-0.5 µA·dec^−1^ *	Ion detection *	Na^+^	10^−6^ M; 10^−6^–10^−1^ M	Na^+^
PEDOT:PSS + Ion-selective membrane	[113]	1.2	0.7	48	47 µA·dec^−1^; 120 mV·V^−1^·dec^−1^	Ion sensing	K^+^, Na^+^	1.5·10^−5^ M; 10^−4^–10^−1^ M	K^+^
PEDOT:PSS + K^+^-selective membrane	[114]		-	414	1035 mV·V^−1^·dec^−1^	Ion sensing	K^+^, Na^+^	10^−4^ M; 10^−4^–1 M	K^+^
PEDOT:PSS + P(T18cr6-ran-EDOT) (Gate)	[115]		10–15	-	49 µA·dec^−1^	Ion sensing	K^+^, Na^+^	10^−4^ M; 10^−4^–1 M	K^+^
PEDOT:PSS + P(T15cr5-ran-EDOT) (Gate)	[115]		11–15	-	37 µA·dec^−1^	Ion sensing	K^+^, Na^+^	2 × 10^−5^ M; 10^−5^–1 M	Na^+^
PEDOT:PSS + ssDNA (Gate)	[116]		0.1	145–149	-	Label-free DNA sensing	ssDNA	10^−12^ M; 10^−6^–10^−1^ M	Hybridized DNA

* These results are achieved in an EGOFET device. *g_m_*: Transconductance. I_D_: Maximum Drain Current. ΔV_g_^eff^: Effectif Gate potential. LOD: Detection limit.
